# Carbon nanofibers based carbon–carbon composite fibers

**DOI:** 10.1186/s11671-023-03944-z

**Published:** 2023-12-21

**Authors:** Nitilaksha Hiremath, Sunay Bhat, Ramiz Boy, Maria Cecilia Evora, Amit K. Naskar, Jimmy Mays, Gajanan Bhat

**Affiliations:** 1 Center for Applied Energy Research, University of Kentucky, Lexington, KY USA; 2 University of California LosAngeles, Los Angeles, CA USA; 3 Fiber and Polymer Science, North Carolina State University, Raleigh, NC USA; 4 Materials Science and Technology Division, Oak Ridge National Laboratory, Oak Ridge, TN USA; 5 Department of Chemistry, University of Tennessee, Knoxville, TN USA; 6 Textiles, Merchandising & Interiors, University of Georgia, Athens, GA USA

## Abstract

Textile grade polyacrylonitrile (PAN) was used as a precursor material for carbon fiber preparation. E-beam irradiated polyacrylonitrile grafted carbon nanofibers were dispersed in polyacrylonitrile solution (dissolved in dimethyl formamide). Carbon nanofibers (CNF) infused polyacrylonitrile solution was wet spun on a lab-scale wet-spinning setup to form 50 to 70 µm diameter fibers with 3.2 wt.% CNF-PAN, 6.4 wt.% CNF-PAN, and neat PAN. Precursor fibers were characterized for thermal, mechanical and morphological properties using various techniques. Drawing the precursor fibers further enhanced polymer chain orientation and coalesced the voids, enhancing tensile strength and modulus by more than 150% compared to those of the undrawn fibers. Precursor composite fibers on carbonization showed enhanced strength, compared to that of pristine PAN fibers, by four times and stiffness by 14 times. The carbon–carbon composite fibers were further characterized with SEM/FIB, XRD and tensile strength. The property improvements were dependent on the uniform distribution of carbon nanofibers, and surface modification of carbon nanofibers further enabled their dispersion in the composite fibers. Furthermore, 3.2 wt.% CNFs in PAN fibers showed maximum improvement in properties compared to 6.4 wt.% CNF in PAN fibers, indicating that the property enhancements go through a maximum and then drop off due to challenge in getting uniform distribution of nanofibers.

## Introduction

Carbon–carbon composite (CCC) structures, e.g. nose cones of missiles and space shuttles, are prepared by infiltrating woven or braided carbon fibers (CFs) with pitch or phenolic resins and carbonized to form CCCs. Pitch or phenolic resin infiltrated CF preform is carbonized in N_2_ atmosphere at 1500 °C [1]. Carbon nanofibers (CNFs), sp^2^ based nanostructures, possess light weight, high strength (≈ 2.9 GPa) and modulus (≈ 240 GPa), high electrical and thermal conductivities [2]. CNFs are used in toughned composites for automotive/ aerospace applications, biosensors, electrodes and supercapacitors, tissue engineering scaffolds etc. [3]. CNFs are reinforced in various polymers, PAN, PVA etc. to make composite fibers, however there is a lack of uniform distribution of CNFs in the polymer matrix due to high surface area of CNFs [3, 4]. They are prepared by CVD technique and the diameter and length of CNFs are ≈ 100 nm and ≈ 200 μm, respectively. As the surface of the CNFs is rough due to truncated structure, the bonding between polymer and CNFs is better than most of the nanofillers [2–4]. Generally, the CNFs are directly dispersed in polymer matrix for composite fiber preparation, however, there are void formation in the fibers [3, 5, 6]. CFs are manufactured by high molecular polyacrylonitrile (PAN) (90% of commercially available CF), and more than 50% cost of CF is due to PAN. As an alternative, to reduce the cost of CF, textile grade PAN could be used to make CF with reduced mechanical properties (highly suitable for automotive, wind energy, and other industrial applications) [7]. In this paper, CNFs are surface modified by grafting PAN via e-beam irradiation and these PAN-g-CNFs (PAN grafted CNFs) are dispersed in PAN solution (dissolved in DMF). Surface modification of CNFs with PAN further enhances the binding spots to increase load transfer between PAN and PAN-g-CNFs [4]. This PAN + CNFs solution is wet spun to form composite precursor fibers and further carbonized to form carbon–carbon composite (CCC) fibers. Furthermore, this investigation also shows the viability of CCC fibers used in any high-temperature applications (e.g. electronic heat sinks) where structural integrity is critical [8]. Electrical characterization data shows that these fibers could be used for electrostatic discharge and RF shielding applications.

## Experimental

Carbon nanofibers (CNFs) (≈ 2900 °C treated) were obtained from Applied Science Inc. Cedarville, OH. Acrylonitrile (AN) (Acros Organics, 99%, stabilized with 200 ppm of hydroquinone), Methanol (Fisher Chemicals)[9]. CNF powder was exposed to electron beam- direct radiation grafting technique (at industrial accelerator operated by NEO Beam – Mercury Plastics, Inc – Middlefield-OH, 3.8 MeV beam energy) in an aqueous solution of AN (20%, v/v in MeOH/H2O) and 4% of inhibitor Mohr’s salt ((NH4)2Fe(SO4)2·6H2O) at pulse current 38.3 mA, 27 kGy/pass with dose rate of 5 kGy/s [4, 9–11]. Resulting PAN grafting on CNF is referred to as PAN-g-CNF henceforth.

Textile grade, low molecular weight PAN (≈ 120,000 g/mol) was used as the polymer matrix, obtained from the Carbon Fiber Technology Facility at Oak Ridge National Laboratory (ORNL), Oak Ridge, TN. DMF (99.9% from Fisher Scientific Co.) was used to dissolve 12 wt.% PAN and disperse PAN-g-CNFs [4, 10]. Dispersion of PAN-g-CNFs in PAN, was achieved by shear mixing for 12 h using a magnetic stirrer and subsequent ultrasonication process for one hour, resulting in a well-dispersed homogenous mixture [4, 10]. Three solutions were prepared: PAN fiber (sample ID – P0), 3.2 wt.% PAN-g-CNF reinforced in PAN fiber (P1), and 6.4 wt.% PAN-g-CNF reinforced in PAN fibers (P2) [4, 10]. All chemical reagents were used as received.

**Fig. 1:**
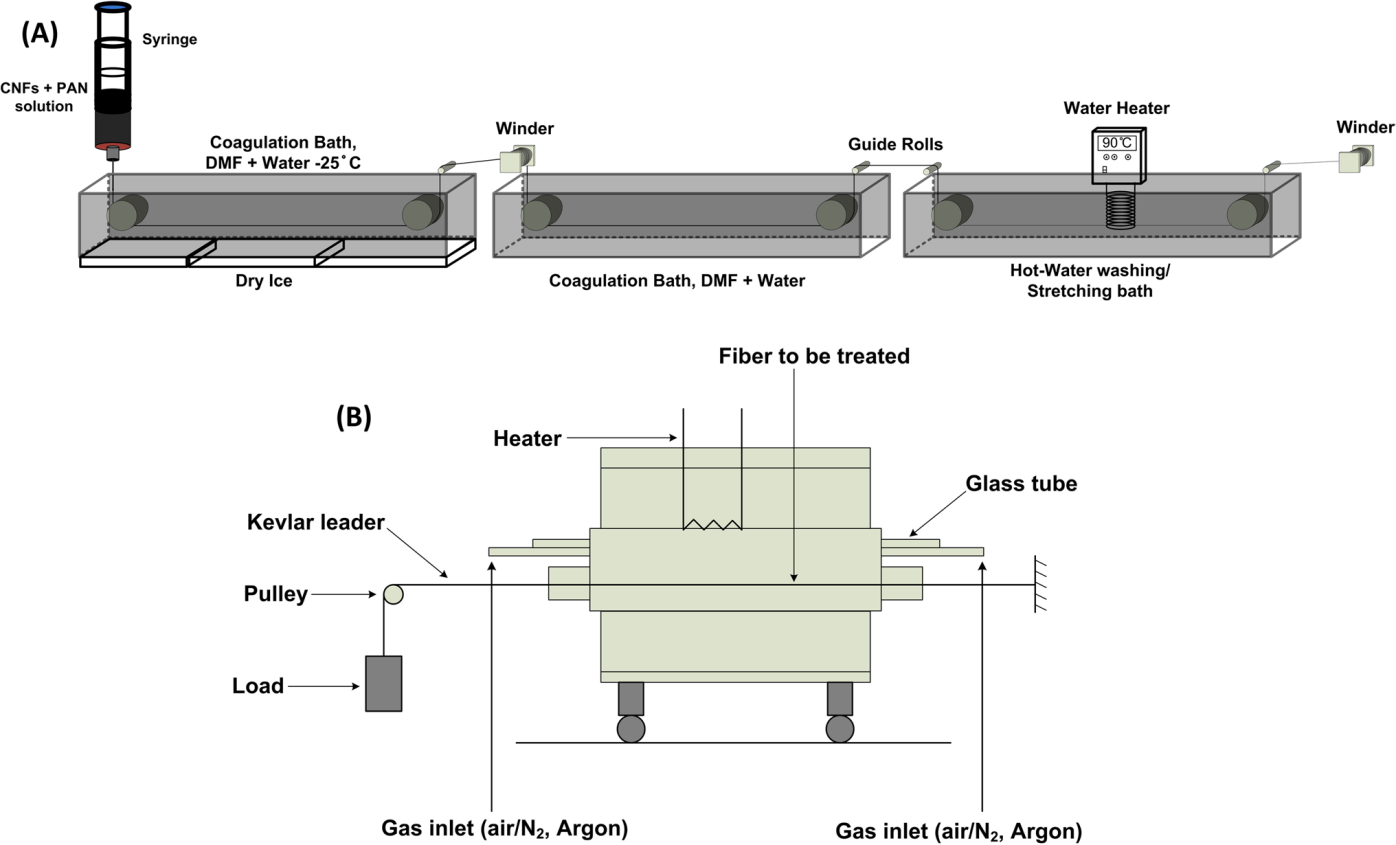
**A** Lab built wet spinning setup for single fiber processing. **B** Lab scale tube furnace used to convert composite fibers to carbon fibers

A home-built solution-spinning lab scale setup was used to coagulate fibers from solutions of the PAN polymer and PAN -CNFs based composite fibers (P0, P1, P2) by wet spinning [10]. From Fig. 1A, a syringe with ≈ 100 μm diameter needle was used as spinnerette and solutions were injected in the first coagulation (water: DMF:: 35: 65 *v/v %*) bath and guided using guide rollers, first coagulation bath was maintained at ≈ − 30 °C. Fiber was further guided to the second coagulation bath (water: DMF:: 65: 35 *v/v %*) maintained at ≈ 25 °C and subsequently to the third coagulation bath (water: DMF:: 100: 0 *v/v %*) maintained at ≈ 90 °C, where most of the drawing and washing of the fiber took place [10]. Total draw ratio of 6.3 was maintained for all of the wet spun fibers. The tube furnace used in this work was made by Applied Test Systems Inc, as shown in the schematic Fig. 1B. Stabilization of precursor fibers were carried out at 100 ml/min air flow and upto ≈ 245 °C, treated for 3.5 h, followed by 100 ml/min nitrogen flow for carbonization upto ≈ 1150 °C, treated for three hours [10]. The temperature and time breakdown for stabilization and carbonization reactions are as follows, 40–230 °C (4.2 °C/min); 230 °C (90 min (isotherm)) to 245 °C (1 °C/min) to 245 °C (60 min (isotherm)) to 500 °C (9 °C/min) to 700 °C (5 °C/min) to 900 °C (5 °C/min) to 1150 °C (6.25 °C/min). The fibers were subjected to a tension of 1 to 1.5 MPa/1000 fibers, throughout the heat treatment process. As the temperature increased from 900 to 1100 °C, the tension on the fibers increased to 3 MPa. Carbonized fibers of P0, P1 and P2 are referred as C0, C1 and C2 here on.

Zeiss Auriga scanning electron microscope (SEM) with focused ion beam (FIB) capability, at the Microscopy Center at the University of Tennessee, Knoxville (UTK) was used in this study and a low working voltage, 3 keV was selected for imaging [4, 10]. FIB ions cut through the sample depending on the acceleration voltage. Once the required cross section is made, SEM is used to analyze the milled surface. MTS single fiber tensile tester, at ORNL, was used to determine the mechanical properties of the PAN and PAN-composite fibers. 25 mm gauge length specimens were tested with a two N load cell at 0.2 mm/min extension rate [4, 10]. Following tensile testing, the fracture surfaces were observed in SEM to examine the failure behavior. PANalytical Empyeran model X-ray diffractometer was used in this study with Cu/K-alpha radiation (wavelength 1.54059 Å) [4, 10]. The X-ray tube was operated at 45 kV, 40 mA. XRD data is shown in Fig. 6. Intensity vs 2θ scans were obtained. Dynamic mechanical analysis (DMA) was conducted, using a DMA RSA3 TA instrument (at ORNL), on neat precursor (P0) and composite precursor fibers (P1 and P2) [4, 10]. A bundle of 10 fibers for each precursor type was mounted on 1.25 cm gauge length card-paper templates. All the 10 fibers were mounted on the card template parallely. The card template was inserted into grips of the DMA instrument and tightened with screws. DMA test on the precursor fibers (P0, P1 and P2) were tested at 10 Hz from – 50 to 180 °C at a heating rate of 5 °C/min. Strain on each specimen was set to 0.5% [4, 10].

**Fig. 2:**
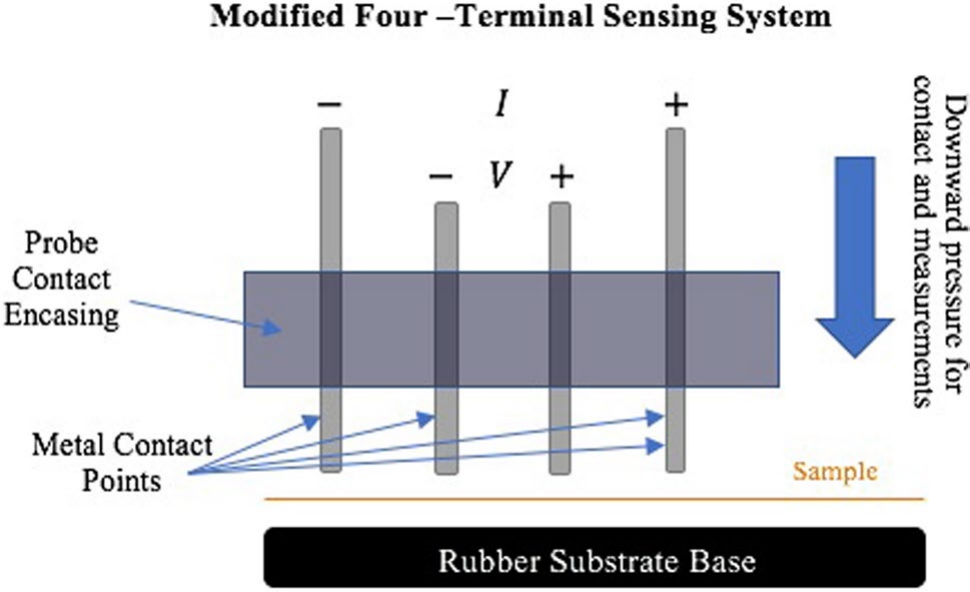
Electrical resistivity measurement setup used in work

Resitivity of the carbonized specimens was calculated from resitance data measured using the Metrohm Autolab PGSTAT204, a laboratory potentiostat/galvanostat. The modular design allows for two to four electrode connections, as shown in the schematic Fig. 2. For this experiment, four connections were utilized creating a four-terminal probe setup, which is often used in measuring sheet or bulk resistance properties of fabricated semiconductors. This method involves applying current through two external probe points and measuring the voltage potential in between the two middle probes. The setup of this probe was customized slightly to accommodate the small size of our fiber bundle sample as well as their fragility. It was observed that standard alligator clips or snaps would stress the sample to the point of breakage, but the system design needed to ensure consistent and even contact for the reliability of measurements. The illustration below displays the measurement system in which four evenly-spaced conductive rods are used with the probe points clipped on to their tops. The rods, housed in a metal encasing but isolated by a rubber spacing, are then placed on the sample which is laid on a cleaned rubber base/substrate. The setup allowed for a decent approximation of bulk resistance values for fiber bundle samples of each specimen. It should be noted that when using this experimental setup, the system was applied only while wearing nitrile gloves, as the metal encasing making contact with bare skin was a potential source of interference.

The accompanying NOVA software was used to setup the experiment. Measurements were made by applying current across the sample with the two outside probes, varying this current from -10 to 10 mA. This was done in steps of about 3 μA, over the course of 40 s. The voltage was polled at a rate of about 330 Hz, or about once every 3.1 ms. The result was a dataset of over 13,100 data points with measured voltage at various applied currents. In order to acquire the resistance, at each data point the voltage was divided by the current for each sample. Since the samples were a bundle of fibers of varying sizes, density of each material was used to calculate the cross-sectional area of each sample after measuring the weight of the bundle. The equation for determining resistivity is:

$$\rho = \frac{R*A}{L}$$

where ρ is resistivity, R is the resistance, A is the cross-sectional area, and L is the length. The length being the measured distance across the two internal voltage probes of the system. This distance was 2 mm. R was obtained by doing a linear regression fit in Excel (method of least squares fit), and utilizing the slope of that line as the best-fit resitance to all the measurement data to minimize error. The cross-sectional area was calculated using sample densities and weights which were cut and measured at the outside length of 4 mm for each bundle and then divided by two to get weight for the 2 mm sample.

## Results and discussion

### Structure and properties of precursors

**Fig. 3:**
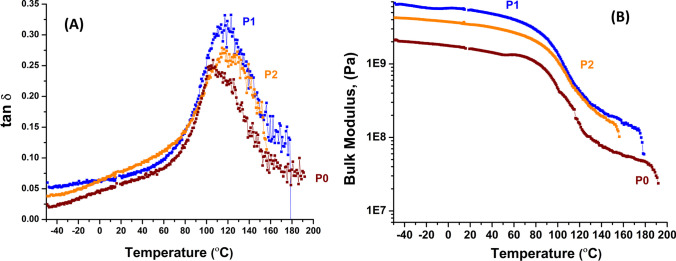
DMA data of composite precursor fibers. **A** tan δ vs temperature of the precursor fibers. **B** Bulk modulus vs temperature of the precursor fibers [4]

**Fig. 4:**
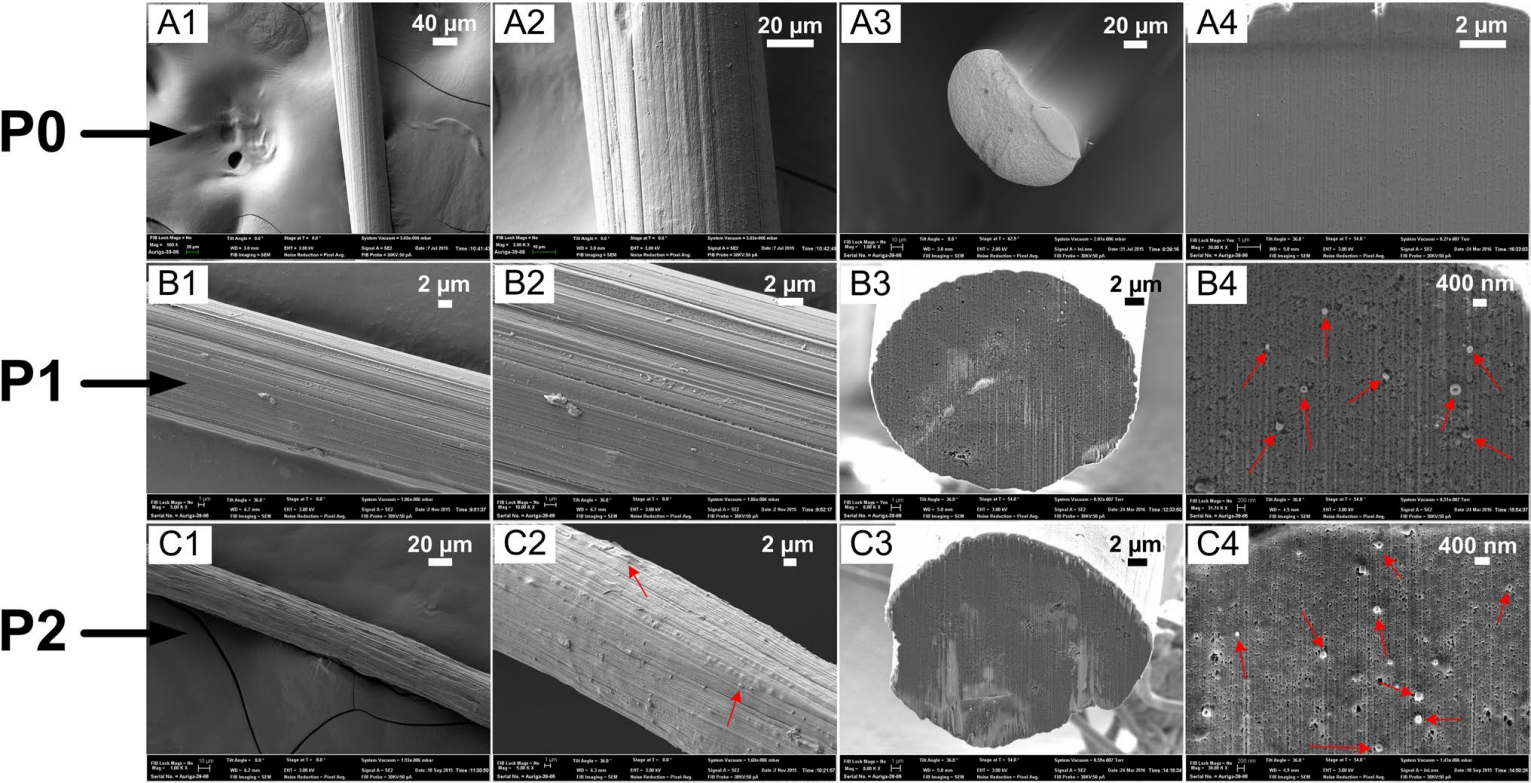
A1, B1, C1 are surface topography SEM images, A2, B2 and C2 are higher magnification surface topography SEM images, A3, B3, C3 are lower magnification cross section SEM images, A4, B4, C4 are higher magnification cross section SEM images of P0, P1 and P2 precursor fibers, respectively. Red arrows in B4 and C4 show the CNFs distributed uniformly. Red arrows in C2 image show the presence of surface level porosity

Figure 3A show DMA data of composite precursor fibers. Mechnical relaxation peak for neat PAN at 105 °C (tan δ, ratio of loss modulus to storage modulus (E˝/ Eʹ)) has increased to 118 °C for composite precursor fibers (P1, P2), showing constrained structure and, hence, rigidization of grafted PAN molecules on CNFs and tan δ values of P0 and P1 are 0.24 and 0.32, respectively [4, 10]. Tan δ value of composite precursor fiber P1, show higher energy dissipation capacity compared to tan δ value of neat PAN P0 fibers [10]. Furthermore, from Fig. 3B, bulk modulus of P1 (6.50 GPa) and P2 (4.23 GPa) show three times and two times higher modulus compared to P0 fiber (2.03 GPa), respectively. Addition of surface modified CNFs in PAN induces restricted PAN molecule mobility and enhancement in modulus due to covalent bond formation between PAN and PAN-g-CNFs [4, 10]. SEM/ FIB images in Fig. 4, A1 to A4 (for P0), B1 to B4 (for P1) and C1 to C4 (for P2), show surface morphology and FIB milled transverse sections of the precursor fibers imaged at low and high magnifications, respectively [10]. As can be observed, there are no visible voids on the tranverse-section of the fibers; however, C2 SEM image (for P2 fibers) show sub-surface porosity as observed by other researchers [5]. Transverse sections of the fibers P0, P1 and P2 do not show porosity validating uniform coagulation, during wet spinning and drawing process, with no visible sheath-core structure, and P1 and P2 cross sections clearly show the uniform distribution of CNFs oriented along the fiber axis.

**Fig. 5:**
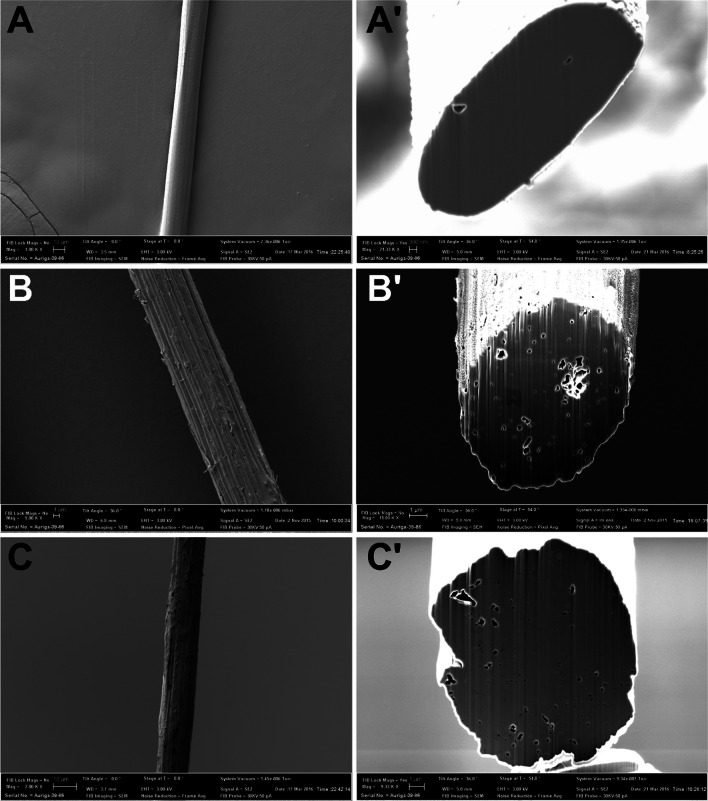
A, Aʹ are SEM images of surface topography and cross section of C0 fibers, respectively. B, Bʹ are SEM images of surface topography and cross section of C1 fibers, respectively. C, Cʹ are SEM images of surface topography and cross section of C2 fibers, respectively

Surface morphology and transverse sections got from FIB miling of carbonized fibers, C0 to C2, are shown in Fig. 5. ‘A’ and ‘Aʹ’ are surface and transverse section SEM images, respectively, of C0 fiber [10]. As observed in Fig. 5 Aʹ no voids were present in the transverse section SEM images. Furthermore, sheath-core effect, observed due to uneven oxidative reactions, was also not observed in the transverse section images [10]. SEM images of surface and transverse sections of carbonized fibers, C1 and C2, are shown in B, Bʹ and C, Cʹ, respectively. As can be observed there are fewer voids and longitudinally oriented ends of CNFs observed in Bʹ and Cʹ [10]. Unifrom distribution and axial orientation of CNFs was achieved and highly oriented CNFs influence significant stiffness enhancement compared to C0 fibers [10].

**Fig. 6:**
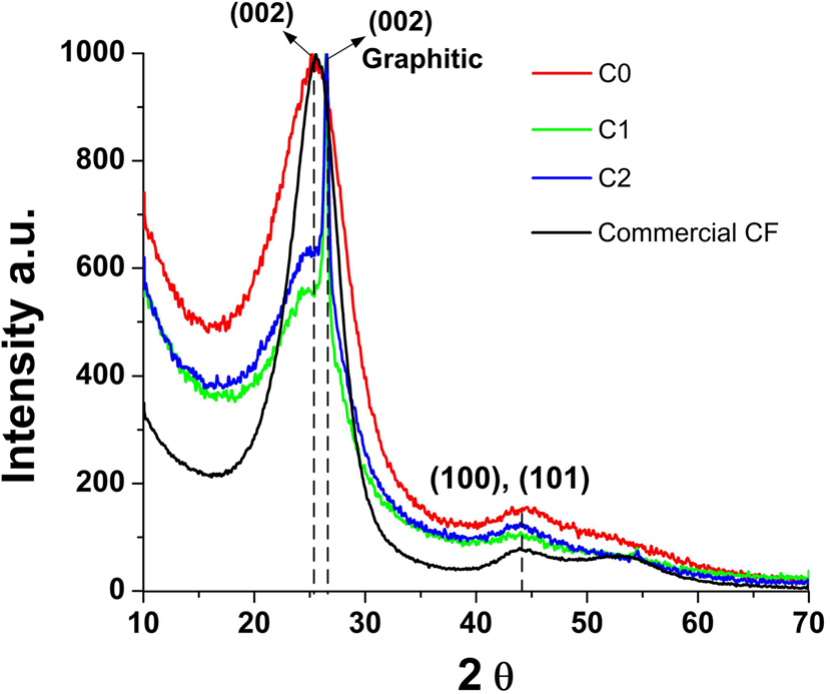
X-ray diffraction C0, C1 and C2 fibers comparing with the X-ray diffraction of commercial CF

XRD analysis of CFs (C0 to C2) compared with a commercial CF are shown in Fig. 6. Evidently, the fibers (C0 to C2) are completely carbonized and the peak at 25.6° which shows the (002) planes. Also, the presence of CNF’s, graphitic structure (002) planes, is visible as a sharp peak at 26.6°. As the concentration of CNFs is higher in C2 fibers the peak intensity is higher compared to that of C1. Furthermore, weak peaks of (100), (101) planes are also visible at 44°. Width of the peaks of C0, C1 and C2 compared to commercial CF show the presence of variation in the graphitic crystal size in the fibers. As commercial CF, generally processed with high molecular weight PAN, are heat treated upto 1500 °C to 1700 °C and the composite CFs, with textile grade low molecular weight PAN, were treated upto 1150 °C there is significant variation in graphitic crystal formation and size distribution. This also shows further optimization is needed in wetspinning of precursor fibers and the carbonization process.

### Tensile testing

**Fig. 7:**
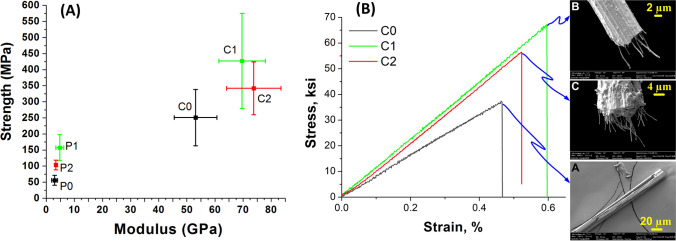
**A** Strength vs modulus data of P0, P1, P2, C0, C1 and C2 fibers. **B** Strength vs modulus data of hot plate drawn C0, C1 and C2 fibers. SEM images show the typical failed ends of the tensile tested fibers with CNFs appearing for C1 and C2 fibers

Figure 7 ‘A’ shows the tensile strength and modulus of all the precursor fibers (P0 to P2), and carbon fibers (C0 to C2). As P0, P1 and P2 fibers contain less to no voids, and due to near parallel orientation of the CNFs along the fiber axis, strength of the P1 and P2 fibers are higher than that of P0 about three times and two times, respectively; whereas the modulus of P1 and P2 are 1.7 times and 1.2 times higher than that of P0 fibers, respectively. Strength and modulus of P2 are 50% and 42% lower than P1 fibers. This could be due to surface level voids in P2 that induce early failure due to stress concentration. During wet spinning, the P2 fibers had slightly rough texture on the surface compared to P0 and P1 (also observed in SEM images, Fig. 4). As the P2 fibers are coagulating, the solvent and non solvent diffusion could be accelerated near the surface creating sub-surface voids. These voids act as stress concentrations leading to failure of the fibers at much lower load than expected. Furthermore, P1 and P2 fibers are drawn further on a hot plate and the voids coleased to enhance packing/ orientation of polymer chains and hence led to increase in strength and stiffness, as reported [5]. Tensile properties of the carbonized fibers show that the CNFs are dispersed uniformaly without causing any agglomeration, and as a result of this, the strength and modulus of the carbonized fibers C1 and C2 are enahnced compared to C0 fibers. Strength and modulus of C1 fiber are almost two and ≈1.3 times that of C0 fiber. Presence of CNFs in C1 and C2 not only increased strength but also modulus as the graphitic structure of CNFs is stiffer than that of the carbon fiber. Similarly, the strength and modulus of C2 are ≈1.4 times higher than that of C0 fiber. Typical stress vs strain curves of C0, C1 and C2 are shown in Fig. 7 and fracture surfaces of tensile specimens where observed in SEM (Fig. 7). Evidently C2 has more CNFs sticking out of the failed specimen than C1 specimen. However, due to higher concentration of CNFs, higher draw ratio could not be achieved to align the PAN molecules to enhance the tensile properties in precursor fibers. Consequently, C2 has lower strain to failure and breaking load compared to C1. Furthermore, breaking energy of C1 is 34.1 J/m^3^ and C0 is 14.4 J/m^3^; C1 abosrbed ≈ 2.3 times higher (more than double) energy than C0 during tensile loading.

### Electrical resistivity

Electrical resistivity of C0, C1 and C2 fibers were evaluated at 20 °C and 65% humidity. Table 1 below summarizes the results of the resitivity calculations from the measured data. Nearly 70% decrease in resistivity was measured in C2 compared to C0 and 27% decrease in resistivty was observed in C1 compared to C0. Non-linear decrese in resistivity was observed in the data as the percentage of CNFs increased in the fibers. CF has a wide range of thermo-physical properties which could be engineered for the desired applications. Higher temperature (> 1800 °C) treatment of PAN based CF in tension induces higher structural ordering (graphitic structure) resulting in enhanced modulus and reduced electrical resistivities [12, 13]. Commercially available PAN based carbon fibers, such as T300, with tensile modulus of 230 GPa, have electrical resistivity of 1.7 × 10^–3^ Ω-cm [14]. However, enhancement in stiffness, and electrical properties are achieved at the expense of tensile and compressive strength in PAN based CF, due to the large crystal grain formation of the highly ordered turbostratic structure [15]. CNFs are treated at ≈ 3000 °C, which makes them highly graphitic in nature. CNFs resist the PAN molecule motion in the precursor fiber drawing step which affects the final mechanical properties of the CFs.

**Table 1:** Electrical property measurement of carbon fibers

	C0	C1	C2
Weight (g)	0.0012	0.0007	0.0212
Density (g/cm^3^)	1.76	1.78	1.8
Length (cm)	0.2	0.2	0.2
Resistivity (Ω-cm)	154.6 ± 0.3	112.3 ± 3.9	47.4 ± 2.4

## Conclusions

Carbon nanofibers reinforced textile grade PAN fibers were carbonized and evaluated for their structure and properties. Incorporation of acrylonitrile radition grafted CNF in PAN resulted in significant improvement in strength and extension to break, especially with a loading of 3.2 wt.% CNFs in PAN. However, with higher loading of 6.4 wt.%, the property improvements were much lower. The increase in toughness observed due to the reinforcing effect of PAN-g-CNF is dependent on uniform dispersion of the nanofibers in the composite fibers. With higher concentration of PAN-g-CNF, achieving uniform dispersion is a challenge, and that is why the observed increase in tensile properties is not as high as for the lower concentration of ~ 3%. SEM photographs of fracture surfaces show the CNFs pulled out showing good bonding and oriented towards the fiber direction. Also, observed electrical conductivity increase is consistent with increase in tensile modulus that is an effect of reinforcement as well as higher molecular orientation. XRD results show complete carbonization reaction with (002) planes at 25.6° of carbon structure and (002) planes of graphitic structure at 26.6°. Further improvement of carbonization process by imparting higher tension on the fibers can lead to further increase in orientation of carbon planes which reduces the width of the XRD peak (comparable to that if commercial CF XRD) and thereby further enhancing the mechanical properties.

## Data Availability

Data sets generated during the current study are available from the corresponding author on reasonable request.
